# “I have never heard of this reproductive health”: young adults’ understanding of the link between sexually transmitted infections and reproductive health in Eastern Cape, South Africa

**DOI:** 10.1080/17290376.2026.2720403

**Published:** 2026-09-28

**Authors:** Nandile Romane, Khumbulani W. Hlongwana

**Affiliations:** Discipline of Public Health Medicine, School of Nursing and Public Health, University of KwaZulu-Natal, Durban, South Africa

**Keywords:** Sexual and reproductive health, STI, young adults, sexual and reproductive health services, sexual and reproductive health literacy, South Africa

## Abstract

**Introduction:**

Sexual and reproductive health literacy (SRHL) enables young adults to make informed health decisions through knowledge empowerment, without which sexual and reproductive health (SRH) outcomes are compromised. This study is apt, given the experimental nature of young adults with risky sexual behaviours, combined with the high burden of HIV and teenage pregnancy in South Africa. We explored young adults’ understanding of the link between sexually transmitted infections (STIs) and SRH in the Eastern Cape, South Africa, so that health educational interventions responsive to young adults can be proposed.

**Methods:**

Using a semi-structured guide, we conducted interviews with seventeen young adults aged 18–24 to achieve data saturation. These young adults were identified through a heterogeneous purposive sampling technique. All interviews were audio-recorded (with participants’ permission), and field notes were taken to complement audio materials. The interviews were transcribed and translated into English before being thematically analysed using NVivo version 15 software.

**Results:**

The findings of this study have provided insights into young adults’ understanding of the link between sexually transmitted infections and reproductive health, including enablers and deterrents to the utilisation of sexual and reproductive health services in Buffalo City Metropolitan Municipality. Three themes emerged from the analysis, including young adults’ STI/SRH knowledge and knowledge gaps, the implications of young adults’ poor STI/SRH knowledge and young adults’ utilisation of SRH services.

**Conclusion:**

Our findings underscore the importance of tailoring health education interventions to effectively address the broad range of sexual and reproductive health challenges affecting young adults.

## PLAIN LANGUAGE SUMMARY

Sexually transmitted infections threaten reproductive health. The broader problem affecting young adults is a lack of knowledge and awareness of the severity of sexually transmitted infections towards reproductive health. This research explored young adults' understanding of the link between sexually transmitted infections and reproductive health, including how their understanding facilitates or hinders access and utilisation of sexual and reproductive health services in the Eastern Cape, South Africa. A reflexive thematic analysis approach was used, where open-ended questions were used to collect data to explore young adults’ (aged 18–24) understanding of the link between sexually transmitted infections and reproductive health. We found that there is a knowledge gap in the link between STIs and SRH among young adults, and it negatively influences their intent to engage in safe sexual behaviour and positive SRH service utilisation behaviour. The findings suggest we should tailor health education interventions that effectively address broad sexual and reproductive health challenges affecting young adults.

## Introduction

Studies focusing on young adults aged 15–24 years have gained traction, given that this population group accounts for 35% of the global burden of disease, mainly sexually transmitted infections (STIs) (Bhana, Jonas, Ramraj, Goga, & Mathews, 2019; Hensen et al., 2024). The World Health Organisation (WHO) posits that this age group is prone to risky sexual experimentation, thus exposing them to STIs, hence the need for sexual and reproductive health literacy (SRHL) in this age group (Bolarinwa & Boikhutso, 2021; Hensen et al., 2024; Ninsiima, Chiumia, & Ndejjo, 2021; Samkange-Zeeb, Spallek, & Zeeb, 2011). Evidence shows that SRHL empowers young adults with helpful health information for shaping their behaviour and health service utilisation (Atuhaire, Ngendakumana, Galadima, Adam, & Muderhwa, 2021; Sørensen et al., 2012). Despite the potential contribution of SRHL, studies on this phenomenon are rare in Sub-Saharan Africa (SSA), with most studies conducted in high-income countries (Amanu, Birhanu, & Godesso, 2023). SRHL should be at the core of promoting Universal Health Coverage (UHC), which is part of the broader agenda towards attaining the 2030 Sustainable Development Goals (SDGs) (Bhana et al., 2019; Ravindran & Govender, 2020b).

There is a need to prioritise investing in young adults’ health by ensuring that intervention strategies are tailored to achieve SDG 3 on health (Bhana et al., 2019; Ravindran & Govender, 2020b). Currently, many young adults living in SSA and South Africa are experiencing a continuous increase in undesirable SRH outcomes, including sexually transmitted infections (Amanu et al., 2023; Bolarinwa & Boikhutso, 2021; Obiezu-Umeh et al., 2021). Regrettably, young adults in many African countries have unmet SRH information needs (Belay et al., 2021). Progress towards achieving UHC for all young adults in South Africa is also slow, which is particularly concerning given that 18.5% of the country’s population comprises young adults (Bhana et al., 2019).

The WHO global agenda to promote young adults’ sexual and reproductive health and rights (SRHR) has been identified in Southern Africa as a priority to achieve UHC and SDG goals 3 on health, 5 on gender equality and 10 on reducing inequalities (Areskoug-Josefsson, Schindele, Deogan, & Lindroth, 2019; Chandra-Mouli et al., 2015; Lynch, Macleod, Chiweshe, & Moore, 2025; Plesons et al., 2019). The realisation of the SRHR strategy promises to reduce adverse health outcomes among young adults by improving the quality of sexual health education and the availability of SRH services (Chandra-Mouli et al., 2015; Lamaro Haintz, Hanna, & Taket, 2023). South Africa is among the Southern African countries that have adopted SRHR policies (Waldman & Stevens, 2015). South Africa has been identified as one of the countries with a high burden of SRHR-related issues, but its SRHR policies are considered among the most progressive globally (Lamaro Haintz et al., 2023; Waldman & Stevens, 2015). A study conducted in the Eastern Cape reported that SRHR issues were primarily related to HIV/AIDS and were construed as shameful, embarrassing and immoral (Lamaro Haintz et al., 2023).

Studies in South Africa have reported that low STI knowledge levels and a lack of adequate sexual health information contribute to the SRH’s undesirable outcomes (Mbizvo & Zaidi, 2010; Mokgatle, Madiba, & Cele, 2021; Mwamba, Mayers, & Shea, 2022). Young adults in the country have unmet needs for efficient, practical education on SRH and STIs. The WHO’s evidence-based intervention indicates that young adults are most likely to make informed decisions when they are knowledgeable about their SRH through Youth-Friendly Health Services (YFHS) (Mokgatle et al., 2021; Obiezu-Umeh et al., 2021). The issue of low STI knowledge is two-fold, broadly including young adults’ poor information-seeking behaviour and low uptake of YFHS as a source of SRH information (Amanu et al., 2023; Atuhaire et al., 2021; Holtman, Bimerew, & Mthimunye, 2023). A cross-sectional study in the rural Eastern Cape reported that adolescents have inadequate STI knowledge, mainly due to the scarcity of reproductive health education among the youth (Tugli, 2012).

There is a need for young adults and the rest of the public to be informed about the adverse effects of STIs on reproductive health, including pelvic inflammatory disease, poor pregnancy outcomes, infertility and genital malignancies, such as cervical cancer and urethritis (Kharsany et al., 2020; Onywera, Mabunda, Williamson, & Mbulawa, 2022; Samkange-Zeeb et al., 2011; Taku et al., 2021). Evidence suggests that having viral STIs, such as *human immunodeficiency virus* (HIV) or *herpes simplex virus* (HSV-2), increases the risk of contracting curable STIs (Kharsany et al., 2020; Onywera et al., 2022; Taku et al., 2021). The Eastern Cape study reported an increase in the acquisition of *human papillomavirus (HPV)* among adolescent girls after early sexual debut (Onywera et al., 2022), and so were curable STIs among young women below the age of 25 years (Taku et al., 2021).

Young adults in South Africa engage in risky sexual behaviour, which leads to undesirable SRH outcomes, including increased risk of STIs (Aventin et al., 2021; Sserwanja, Mwamba, Poon, & Kim, 2023), with the common risky sexual behaviours being multiple sex partners, early sex initiation, unprotected sex and transactional sex (Amanu et al., 2023; Bolarinwa & Boikhutso, 2021; Essack, Toohey, & Strode, 2016; Jonas et al., 2020; Mwamba et al., 2022; Sserwanja et al., 2023). The risk factors include alcohol use, negative peer influence and lack of SRH knowledge (Amanu et al., 2023; Bhana et al., 2019; Mokgatle et al., 2021; Ninsiima et al., 2021; Onywera et al., 2022; Selikow, Ahmed, Flisher, Mathews, & Mukoma, 2009). Understanding these risk factors is crucial for health policymakers to design effective interventions (Kyilleh, Tabong, & Konlaan, 2018; Mokgatle et al., 2021) that extend beyond knowledge acquisition.

Locally relevant, youth-friendly health services responsive to the needs of young adults are needed to address unmet SRH needs (Jonas et al., 2020; Mayeye, Lewis, & Oguntibeju, 2010; Ninsiima et al., 2021; Obiezu-Umeh et al., 2021; Ravindran & Govender, 2020a). In SSA, inadequate SRH information and negative attitudes of healthcare workers (HCWs) deter young adults from accessing appropriate and effective sexual and reproductive health services (Ninsiima et al., 2021; Ravindran & Govender, 2020a), including long waiting times for SRH service, confidentiality concerns in consultation rooms and a lack of support from family (Belay et al., 2021; Bhana et al., 2019; Bolarinwa & Boikhutso, 2021; Essack et al., 2016; Holtman et al., 2023; Kyilleh et al., 2018; Mbizvo & Zaidi, 2010; Ninsiima et al., 2021; Obiezu-Umeh et al., 2021). On the other hand, knowledgeable healthcare workers with a positive attitude promoted reproductive health and utilisation of SRH services (Bossonario et al., 2022; Jonas et al., 2020).

Reports suggest that South Africa experiences a continuous increase in adverse SRH outcomes, such as STIs and unplanned pregnancies among young adults, and 55% lack adequate knowledge about the risk of STIs and becoming pregnant (Bolarinwa & Boikhutso, 2021; Holtman et al., 2023). Therefore, there is a need to explore young adults’ understanding of the link between STIs and SRH in South Africa, particularly in the Eastern Cape Province, especially given the province’s highest STI incidence rate (Frank et al., 2023). Efforts to find studies exploring young adults’ understanding of the link between STIs and SRH were unsuccessful with respect to South Africa. This study explored young adults’ understanding of the link between STIs and reproductive health in Buffalo City Metropolitan Municipality, Eastern Cape, to inform the development of SRH education interventions responsive to their needs.

## Method

### Study design

We used a reflexive thematic analysis to inductively explore young adults’ understanding of the link between STIs and reproductive health in selected healthcare centres in the Buffalo City Municipality, Eastern Cape province (Braun & Clarke, 2021).

### Study setting, sampling and participants

This qualitative study was conducted in three community healthcare centres (CHCs) that offer Youth Friendly Health Services (YFHS) in Buffalo City Municipality (BCM), Eastern Cape, South Africa. These CHCs included Duncan Village CHC, Nontyatyambo CHC and Mount Coke CHC, reflecting a rural-urban mix to capture perspectives in different contexts. Using a purposive heterogeneous sampling strategy, we recruited self-reported sexually active young adults aged 18–24 years attending the YFHS unit in participating CHCs. The researchers had no prior relationship with the participants. The purposive heterogeneous sampling enabled us to recruit a demographically diverse group of participants. The data saturation, at which point no new information was obtained, was reached in the seventeenth in-depth interview (Žydžiūnaitė & Tauginienė, 2017).

### Data collection

The lead female author (a pharmacist with a B.Pharm degree and at final stages of Master of Public Health studies) conducted in-depth face-to-face interviews in a private room at the community health care centres using a semi-structured interview guide. The participants may have been aware of the lead researcher’s role as a healthcare worker but not her personal goals and aspirations. There were no non-participants in the room. Interviews were conducted only after participants had consulted with nurses and doctors while waiting for their treatment at the dispensary. The lead author worked closely with the nursing staff to minimise patient inconvenience. All potential participants were provided with study information and signed informed consent forms before participating in the interview. No potential participant refused to participate in the study. Participants had the option to be interviewed in either English or isiXhosa, with six opting for isiXhosa. Interviews lasted approximately 40 min and were audio-recorded (with participants’ permission) and complemented with hand-written field notes. The interview questions were not pilot-tested; however, they were improved several times following input from the senior author. No repeat interviews were conducted. The seventeen interviews were conducted between 10 May and 22 May 2024.

### Data analysis

Data collection and analysis were conducted iteratively to determine data saturation, which was reached at the 17th interview (Braun & Clarke, 2012, 2021). The lead researcher transcribed the recorded interviews verbatim before translating them into English, a process verified by the senior author. Data analysis was guided by Braun and Clarke’s six-phase reflexive thematic analysis process, with assistance from NVivo 15 software (Braun & Clarke, 2012; Byrne, 2022). The process began with familiarisation with the data by repeatedly reading the transcripts. Under the guidance of the senior author, the lead author generated and reviewed the initial codes and themes to identify the shared meaning (Braun & Clarke, 2012; Byrne, 2022). The data analysis was conducted inductively to allow themes and subthemes to emerge from the data. No participant was checked to provide feedback on the findings.

### Measures to ensure the trustworthiness of the study

We followed Lincoln and Guba’s (1985) four principles – namely, credibility, transferability, dependability and confirmability – to enhance the trustworthiness of the findings (Stahl & King, 2020). The audio-recorded interviews were transcribed verbatim and supplemented with handwritten field notes, providing a detailed description of the study context. The lead author had virtual debriefing sessions with the senior author after each round of interviews. The lead author, as a healthcare professional and having completed the Master of Public Health coursework, including Qualitative Research Methods as a core module, used a reflexivity research journal to mitigate her biases and assumptions about the research process and outcomes (Stahl & King, 2020).

### Ethics approval and consent to participate

The study obtained ethics clearance from the Biomedical Research Ethics Committee (BREC) of the University of KwaZulu-Natal (UKZN) on 17 November 2023 (Reference number BREC/00006199/2023). The study was adequately explained to all potential research participants before they voluntarily signed the informed consent forms, thereby allowing them to participate. Participants were assigned codes to hide their identities throughout the data collection and analysis.

## Findings

We interviewed 17 participants aged 18–24, most of whom (*n* = 14) were female, and their educational levels ranged from Grade 11 to tertiary qualifications (Table 1).

**Table 1.** The participants’ demographic characteristics.

Participants	Age	Gender	Education	Employment status	*Health facility for recruitment
P1	18	Female	Grade 12	Information not provided	DV CHC
P2	22	Female	Information not provided	Employed	DV CHC
P3	24	Female	Information not provided	Employed	DV CHC
P4	18	Male	Grade 12	Information not provided	DV CHC
P5	20	Female	Information not provided	Employed	DV CHC
P6	23	Female	Information not provided	Employed	DV CHC
P7	23	Male	University	Information not provided	DV CHC
P8	23	Female	Information not provided	Unemployed	DV CHC
P9	23	Female	Information not provided	Employed	DV CHC
P10	20	Male	University	Information not provided	DV CHC
P11	20	Female	Information not provided	Unemployed	MC CHC
P12	18	Female	University	Information not provided	MC CHC
P13	18	Female	Grade 12	Information not provided	MC CHC
P14	18	Female	Grade 12	Information not provided	NCHC
P15	18	Female	Grade 12	Information not provided	NCHC
P16	19	Female	Grade 11	Information not provided	NCHC
P17	18	Female	Grade 11	Information not provided	NCHC

*DV CHC – Duncan Village CHC, MC CHC – Mount Coke CHC, NCHC – Nontyatyambo CHC.

*Information not provided – It was not collected from the participants.

Data analysis produced three themes and five sub-themes, namely: (1) young adults’ STI/SRH knowledge and knowledge gap (knowledgeability of young adults about STIs and SRH, and the importance of protective measures in SRH); (2) implications of young adults’ poor STI/SRH knowledge (risky sexual behaviour) and (3) young adults’ utilisation of SRH services (deterrents to young adults’ utilisation of SRH services, and enablers to young adults’ utilisation of SRH services) (Figure 1).

**Figure 1.**
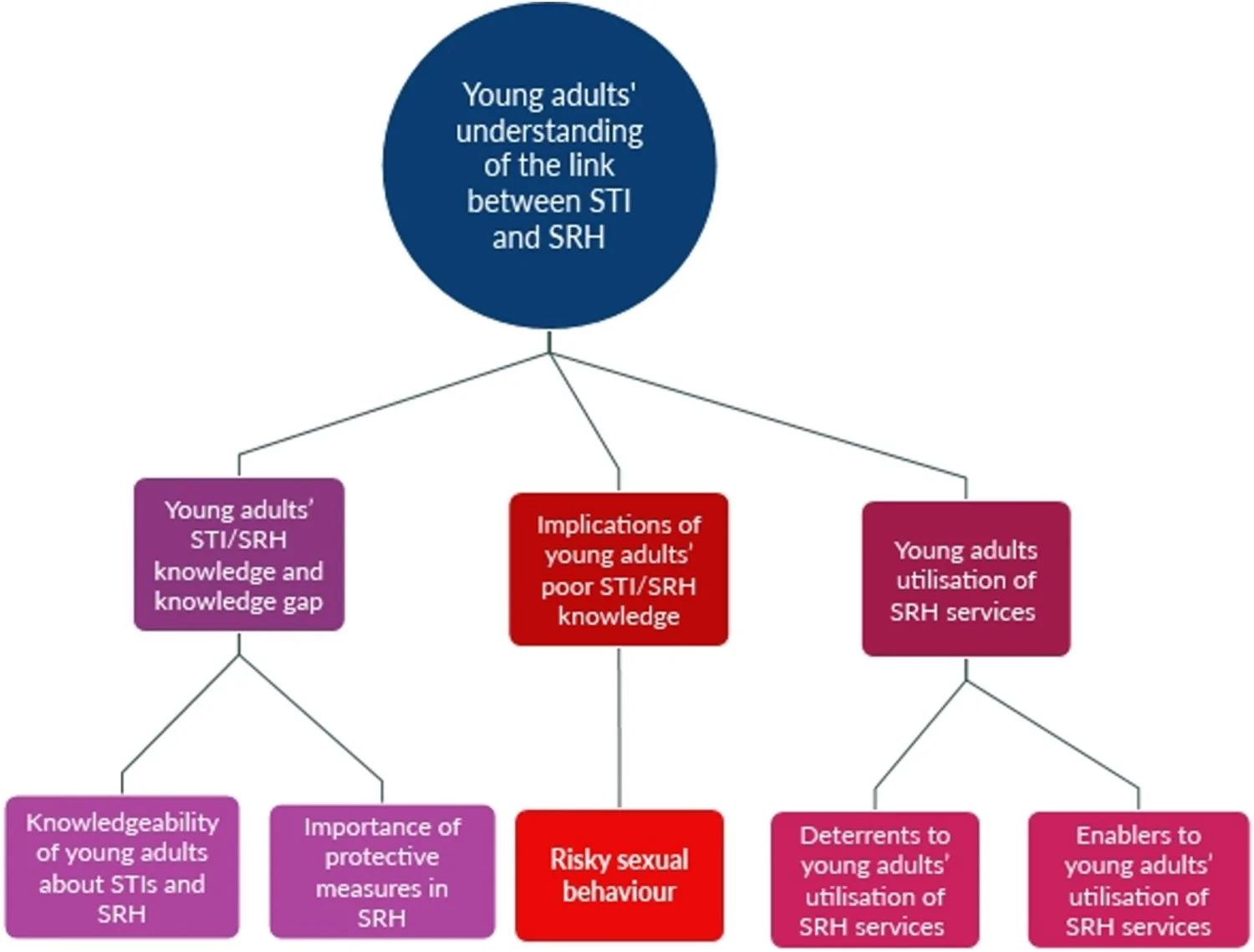
Illustrative diagram of themes and sub-themes.

## Young adults’ STI/SRH knowledge and knowledge gap

The first theme of the study describes what the young adults knew about STIs and SRH, including the gaps in their understanding of the importance of protective measures in SRH. The findings showed no clear educational, gender or age patterns in participants’ knowledge of STIs, SRH and protective measures.

### Knowledgeability of young adults about STIs and SRH

While participants demonstrated some general knowledge about STIs, their symptoms and protective measures (P1–P17), none accurately specified any specific STI (P1–P17).

STI, it’s a drop, right? If it’s a drop, I have heard it’s painful … But I have heard you get it when you sleep with a girl, and she will also. Because they say there are different types of STIs, you see. P7

Well, let me say then, I know a few symptoms of STIs. Let’s say you have just had sex with someone, and then you get itchiness under there or have pimples under there. Those are the ones I know. Others? Oh yes, when you have a burning sensation when you pee. I have forgotten about others. I think also when you get a dirty discharge, I think those are the ones I remember … P11

I don’t know, really … but there are a few I’ve learnt about in high school: your gono-something and your syphy-what-what. But I think some will affect you more than others, like me. I consider myself to have an STI, although I don’t know why the doctors are not diagnosing it. So, I think one is less than for other people’s … P10

The research participants showed no understanding of STI pathogenesis but thought that untreated STIs could evolve into HIV infection (P5, P8, P13). One participant (P5) thought the infection was transmitted through using an unwiped toilet seat with urine contamination from previous users. In contrast, the other participant hesitantly thought that an STI could turn into HIV (P13).

Maybe the STI can turn … well, I'm not sure about this … but maybe the STI will turn to HIV, and the person ends up HIV positive since they didn't treat the STI …  P13

Oh, and another thing, I stay in a rented flat. We share a toilet with other tenants, so a guy there works for Bolt – he comes home with a new girlfriend every day! Everyday! I’m unsure whether I got a toilet infection from them … P5

One participant made a vague link between STIs and SRH.

Ok, so STIs, which are Sexually Transmitted Infections and then the reproductive health, which has to do with giving birth, right? Ok, so I would say STI affects the person – you know, when I was doing my research about what’s making me sick … I was looking at some pictures there, and I could see that your reproductive organs would get rotten and have gangrene, you see. So, I think they are closely related – I think when someone has an STI, then reproductive organs will be affected badly. P10

Despite most participants (P1, P3, P5–P7, P9–P17) reporting knowledge of at least one STI symptom, few (P2, P4, P8) could not name any symptom.

STIs cause a very smelly discharge, so I don’t think there is anyone who would leave an STI untreated … and it’s very painful to pee when you have an STI. So, I think the person pushes themselves to go to the clinic … P12

All the participants (P1–P17) showed a lack of knowledge about the implications of STIs on reproductive health, especially the reproductive health concept. When asked a specific question about the consequences of STIs on pregnancy, they either responded with ‘I don’t know’ or provided vague answers. One participant (P12) attributed her lack of knowledge to not having studied Life Sciences in school.

I have never heard of this term [reproductive health] … It’s my first time hearing about it. P11

My problem is that I have never studied life sciences as a subject. So, I don’t know everything you are talking about. I honestly don’t know about reproductive health. P12

The findings revealed that there is a knowledge gap in the link between STIs and reproductive health. The participants reported that they are unaware of the consequences of contracting STIs during pregnancy (P2, P5–P6, P8–P9, P11–P14, P16–P17). Only one participant (P10) admitted that young adults were clueless about SRH. He thought the baby might be born with Down syndrome because of STIs.

Maybe the baby you give birth to will have Down syndrome. I don’t know what causes Down syndrome. Maybe it might increase the chances of your child having Down syndrome. I don’t know. It’s just my way of thinking. P10

All participants (P1–P17) provided vague answers regarding the implications of leaving STIs untreated.

I think if someone left an STI infection untreated, they would get sick … definitely, they would get sick. Who can endure something that is burning them? Definitely, I think it would make them sick. That can’t be normal. If they don’t treat it in time, it will definitely make them sick … P17

Maybe they will end up being damaged on their vagina, and when they are having sex with someone, that person will have to use a condom. P15

*What I know about an STI on a reproductive organ is that it can cause it to fall off * … *like the penis can fall off. That means it won’t function as a reproductive organ. That is all I know*. P7

### Importance of protective measures in SRH

All participants (P1–P17) knew that condoms are used to protect one from STIs and pregnancy; some (P3, P7, P8) believed dual methods are vital because contraceptives do not protect one from sexually transmitted infections.

Ok, the condom, you must use it to protect yourself from diseases like STIs and HIV … and then contract-something … what do you call it? But prevention, yes, is also important when you don’t want to have a child. But you must also use a condom while you are using the prevention injection or pills … P3

And also, she would advise me to condomize because the condom doesn’t only prevent pregnancy … but also STIs and STDs. P8

Some participants (P5–P8, P14, P17) stated that condom use is not a must if the other one is on a Pre-Exposure Prophylaxis (PrEP) regimen, on contraceptives or in known HIV positive/unknown HIV status.

So, it’s important to suggest PrEP to a girl if she’s not comfortable with a condom. I am willing to compromise my condom only if she’s willing to take PrEP because it will also help them. P7

When we met, we didn’t use it [condom], but there was a time I was late for my injection … then I said to him we should use it. P8

You have to use a condom when you go to sleep with someone if you don’t know their HIV status. If you are not going to use a condom, you must know their HIV status … . P14

This participant (P12) shared that, in her opinion, people do not care about HIV as well; they mainly care about preventing pregnancy.

I think the issue is using the contraceptives – so the person would … I think HIV is not taken seriously; that’s the problem. What is taken seriously is that they will not become pregnant. Once a girls use contraceptives, they thinks they will not fall pregnant, so there is no need to use a condom, so they do not care about HIV. P12

## Implications of young adults’ poor STI/SRH knowledge

The second theme of the study shows that young adults’ poor STI/SRH knowledge can potentially lead to risky sexual behaviours.

### Risky sexual behaviours

Our findings also revealed a range of risky sexual behaviours that participants confessed to either engaging in themselves or practising by their peers. These risky sexual behaviours included early sexual debut (P1–P5, P7, P10, P14), inconsistent condom use (P2–P9, P11, P13–P17) and having multiple sexual partners (P11, P14–15, P17). Most of the participants (P1, P4–P7, P12, P14–P15) initiated sex when they were below 16 years old. Our findings revealed that the practice of multiple sexual partners was pervasive in this age group.

I once had an STI before … an ex-boyfriend of mine cheated on me … It was that sort of thing; he slept with someone during the day, and then I also went in the night to sleep with him. It was something like that. And then I had an STI like that … P5

Maybe I would’ve gone to another boyfriend that is not mine … or if my friend also went to see another boy that is not hers. So, we will tell each other to go to the clinic to get tested for HIV and pregnancy. P11

Other risky sexual behaviours included casual sex when they were out drinking with friends.

I wouldn’t have used the condom. Like I use a condom if maybe I go out to a club and then maybe I meet someone there that I don’t know, and they also don’t know me, and maybe when I come back from him [new guy] the following day, I will never see him again. Or maybe if I meet a friend with benefits when I go out, then I will use a condom. But if I have an official thing with someone, like I would say I am dating that person, I don’t use a condom. P15

At times, young adults anticipate casual sex and carry protection, especially when they go out with friends.

She asked why I use PrEP? … and I told her that it’s a backup plan, just in case something goes wrong at night when I am out with friends … so that nothing happens to me where I get HIV. P17

Drunkenness also drove casual sex.

It was not with someone I was seeing … like, we had a small party in our res. And then I got drunk, and I got a partner there at that party, and then I slept with her. Then that was it. I have seen her around, but have had no conversation with her. When I hear about her, she is someone who has multiple partners. P10

Some participants (P6, P8–P9, P12) believed that a long-term relationship was a good enough reason to waive condom use, and they made no mention of HIV testing before waiving condom use. Secondly, the link between condom usage and unfaithfulness disempowered those who insisted on consistent condom use. The female participants (P1, P9, P11) indicated that they do not have the power to raise their desire to use a condom in a relationship because of the fear of being seen as unfaithful and eventually getting dumped by their partners.

Well, I trusted him too much because I was dating him for two years … so, I was like, there is no need for it. At the same time, I am on contraceptives, so there was no need for me to use a condom. Well, I didn’t even expect the STI because I didn’t have it for two years … And then in 2022, when I got infected, that’s when I drew the line and decided we are over. P5

Hey, I don’t know why we stopped using it, either … It’s that man thing where they just don’t want to use a condom anymore, and he would be arguing that we have been dating for a while, so what’s the point of using one? We will use condoms forever, as he told me, things like that. He would say if I wanted a condom, then it meant there was someone else I was sleeping with without a condom, things like that. I would suggest we use a condom, but he would brush it off by saying that maybe I cheated, that’s why now I want us to use a condom. P9

The social norms and social interactions were reported as drivers of risky sexual behaviour.

Fortunately for me, I grew up with my uncle, and he’s not older than me … how old am I now? I’m 23, and he’s 30. So, he is 7 years older than I. The kind of talk I got from him was to encourage me to have sex, and he would say, you must stick it in my nephew. Like, get it, my nephew, with girls, you must have sex with the girls and make sure you don’t fall in love with them. So that’s the kind of motivation I got from my family sexually. Then it was up to me to decide what to do with my life. P7

I didn’t say no because I wanted to experience sex without a condom, too; my friends always told me about it without a condom. Most of the girls, when they are sitting together, are always talking about sex, so I wanted to experience it as well. P16

The findings also revealed that the use of condoms was affected by negative attitudes towards them, incorrect condom use and their availability (P3–P4, P7–P8, P12, P14–P15, P17).

I have one partner, and we decided not to use it since we don’t enjoy sex when we are using a condom … . P8

When we met, we didn’t use it … But there was a time I was late for my injection, and then I said to him we should use it. But I feel like both of us could feel that; well, I don’t know whether he had put it on the wrong way or what, but I could feel that he had a condom on. So, he suggested taking it out – and I was fine with it. Ever since then, we have never used it again. P8

I would say I do use the advice, but sometimes I don’t use a condom. Maybe the condom is not there, or you didn’t think about it. P4

## Young adults’ utilisation of SRH services

The third theme showed that while some factors enabled young adults to utilise SRH services, others deterred the uptake. HCWs, family members, friends, and young adults themselves either deterred or enabled the utilisation of SRH services.

### Deterrents to young adults’ utilisation of SRH services

Participants (P1, P3, P5, P8, P12–P13, P15) identified two main deterrents to their utilisation of SRH services: fear of compromised confidentiality arising from HCWs being from their neighbourhoods and HCWs’ attitudes and judgment.

The problem with the clinic where I stay is that most of the nurses there stay around in our area … so patients’ news gets spread all over the location. You’d hear that so-and-so doesn’t take their pills. So, it was better to use this clinic since I don’t stay around here. P12

Because they shout at us … Yhoo! They shout at us in shame. And some kids will be scared to come to the clinic, for instance, for contraceptives, because they know they sleep with boyfriends sometimes. But when they come here, they’ll be told, “Why so young, you already sleeping with boys?” … I wish the nurses could stop that. P3

Young adults also have their demographic preferences regarding HCWs. The participants (P3, P7–P8, P13, P15) expressed a preference for being consulted by younger healthcare workers of the same gender as themselves, as they would not be judged.

Nurses to be younger – because older nurses … No! They don’t make it easy for youth to visit the clinic. P3

It is still a challenge, especially when a female nurse asks questions instead of a male nurse. You get that thing inside that says, ‘Oh, no, sister, how can you ask me something like that?’ It should be more welcoming to the guys and create an environment where males can access the services offered by the clinic. To help men as well, create a platform for discussing matters like STIs by hiring a male nurse. And some guys might not want to come to the clinic to talk about their private parts if they know a female nurse will help them. P7

The participants (P10, P12, P16) expressed a lack of confidence in HCWs’ ability to diagnose and treat them, and one participant even suggested that diagnoses be made by an artificial intelligence application that could diagnose their symptoms. The findings indicate that the HCWs lack adequate SRH knowledge.

No, sometimes I don’t feel it’s helpful because I tell you my problems, and you tell me you don’t know what is happening. I expect more, and I expect more. In Grade 12, I had to decide between studying to be a doctor and pursuing a career in electrical engineering. Of course, I chose electrical engineering. But I am disappointed in doctors; I just expected more. So, I was thinking: what if I make an app that lets a person enter their symptoms, and then diagnoses the possible illnesses for each individual? I don’t know – it’s what I feel. I expect more from doctors and nurses, and they have disappointed me. Maybe technology can do far better than these people. I don’t know. Don’t take me wrong, but that is what I think – it came to my mind that maybe we need something like that. P10

Other YFHS-related factors included a lack of privacy in consultation rooms and long waiting times (P6, P8–P9, P13, P15).

Sometimes, when the nurses have gone to lunch, they don’t return early. Sometimes we would end up going home without receiving the help we needed. Sometimes, as I’ve already mentioned, I get irritated when you come here and may be unsure whether you will get help because there is no nurse available to assist you. We can only come here after 2 pm, as we start our study session then. The school allows us to come to the clinic before the study … P13

… hm … maybe make the consultation more private because they can ask you questions while others are around. So, make it private because maybe you don’t want your things to be known by others, like in a private room like this one, so that there won’t be people interrupting you while you are talking. P8

The young adults’ discomfort in discussing sexual issues with elderly family members and misleading information from ill-informed peers also deterred young adults from utilising SRH services (P2, P7, P11, P16).

Because I am scared to tell her [mother] about boyfriends. And be talking to her about those things. She is not someone who is, what can I say? She is not too strict – she is quite open. I can speak to her about anything, but the problem is with me. I’m just not comfortable talking to her about boyfriends. P11

OK … some friends say it’s not good to use an injection when you don’t have a child yet. You can use it when you have a child. What I want to know is whether it is ok to use an injection when you don’t have a child yet. Does it cause infertility at all? P2

Participants also reported financial and structural deterrents. These deterrents included a lack of transportation fare and long distances to health facilities (P10-P11, P8).

… But when I had the symptoms again, I didn’t have the money to come to the clinic. I don’t stay around here. It’s far here – I cannot just come alone. I always need to ask someone to accompany me to the clinic. I can never walk here alone. The times when I don’t come, like the times you find that I have an asthma attack, and there is no money for me to come to the clinic. Then I would ask for an asthma inhaler from my aunts … P11

### Enablers to young adults’ utilisation of SRH services

Young adults viewed the availability, accessibility, and efficiency of SRH services as enablers of their uptake (P1, P5–P6, P9, P11–P13). Support from family and friends (P1–P17) and the availability of SRH information from the community help young adults seek SRH services (P3–P6, P8–P11, P13–P17).

She [nurse] helped me. She said it’s not normal to have menses for a long time. She gave me these pills and said that I should drink all of them in the order they are taken. And then if they run out, I should come back to the clinic, P13

I would say this [YFHS] Unit is fine. There is nothing that delays. I didn’t wait too long to be seen by the nurse. It’s not like when I was on the administration side. Yes, I would also say that the nurses are friendly, just like you. “laughs” … I did come for a fever – but she said they found an STI in my urine P1.

I stay at Kuni, yes, and I clinic here at Mount Coke – but I prefer to come here because the services are good and quick … and it’s never full of patients. This clinic has supported me from day 1 since I was diagnosed with HIV. So, I only come here every 3 months for my 3 months’ supply of ARVs, and then when I have my checkups. P12

Our findings showed that HCWs and the YFHS department were essential enablers of SRH service utilisation. For example, providing health information by healthcare workers using YFHS proved helpful to young adults (P3–P6, P9, P11, P13, P15, P17).

The nurse here is very open – you can come to her whenever you want to know something. For example, what is used for prevention, like the injections? I asked her if you use the injection without using a condom, can you still get pregnant? P13

Participants also mentioned supportive family and friends as enablers of consistent use of SRH services (P1–P17).

Well, when I was young, when I was young, my family told me to go to the clinic to prevent pregnancy … I think I have first-hand support in seeking sexual health, which I received at home. But in terms of taking it deeper into sexual health, not much. Because things like STIs and STDs, I was not aware of them. P8

Participants reported that access to SRH information from schools, community outreach programs, families, and the media enabled them to seek SRH services (P3–P6, P8–P11, P13–P17).

From school … at school, they teach us about those things [SRH] when doing Life Orientation. They warn us about those things because our age now says we must know about them. P14

I mean, we could get a brand that promotes that teaching … like we once had Love Life as well that taught us about preventing HIV and explaining about HIV. P8

I think the best way is to teach through social media. Because a lot of youth now use social media. P10

## Discussion

The findings of this study have provided insights into young adults’ understanding of the link between sexually transmitted infections and reproductive health, including enablers and deterrents to the utilisation of sexual and reproductive health services in Buffalo City Metropolitan Municipality. Three emerging themes included young adults’ STI/SRH knowledge and knowledge gap, implications of young adults’ poor STI/SRH knowledge and young adults’ utilisation of SRH services.

Young adults have been shown to have some general knowledge of STIs but little to nothing on sexual and reproductive health, including the link between STIs and SRH, as well as the implications of STIs on reproductive health. All the participants indicated that they had never heard of the term sexual and reproductive health. Given that hearing about sexual and reproductive health foregrounds the understanding of how the two interact, it was not expected that participants would know about the implications of STIs on reproductive health and pregnancy if they had never heard of reproductive health before. SRH knowledge is essential for improving young adults’ health-related decision-making and uptake of SRH services (Amanu et al., 2023; Atuhaire et al., 2021; Kyilleh et al., 2018; Sørensen et al., 2012). Studies have reported that low STI knowledge levels and a lack of adequate sexual health information contribute to the SRH’s undesirable outcomes, including contracting STIs (Mbizvo & Zaidi, 2010; Mokgatle et al., 2021; Mwamba et al., 2022).

Our study’s findings suggest a lack of understanding, knowledge and awareness of STIs and SRH among young adults. A majority of our participants could name more than one symptom of STIs, concurring with a study in the Eastern Cape, South Africa, where young adults were knowledgeable about STI symptoms (Tugli, 2012). A systematic review conducted in Africa concluded that young adults had general knowledge of the existence of STIs, including STI symptoms, reproductive health implications and prevention methods (Badawi et al., 2019).

Also, participants showed knowledge of STI transmission, and they all knew that condoms are used to protect oneself against STIs. However, they admitted that they used condoms mainly to protect against pregnancy rather than to prevent STIs, consistent with what has been reported in the literature (Samkange-Zeeb et al., 2011). Similarly, a systematic review incorporating twelve studies conducted in SSA reported that young adults’ intentions to use a condom are to avoid future socio-economic consequences, including delay in education, rather than to prevent STIs (Aventin et al., 2021). However, in the same systematic review, two studies reported that they attributed their condom use to their value in preventing STIs and unintended pregnancy (Aventin et al., 2021).

Strangely, our participants’ educational levels did not affect SRH knowledge, contrary to the literature (Belay et al., 2021). Similar studies in Zambia and South Africa reported that low educational levels among young adults are associated with engaging in risky sexual behaviours because they lack the confidence to engage in meaningful sexual health conversations with their partners and parents (Hendriksen, Pettifor, Lee, Coates, & Rees, 2007; Sserwanja et al., 2023). A study conducted in SSA found that few young adults have had the opportunity to access SRH education programmes in schools (Amanu et al., 2023). Meanwhile, our findings have shown that young adults have had the opportunity to access SRH education in schools primarily through the subject of life orientation (LO).

The findings from this study showed that young adults engage in risky sexual behaviours, mainly due to inadequate SRH knowledge and low-risk perception of contracting STIs. Our study revealed pervasive early sexual debut, with many of whom started sex below the age of sixteen years and were exposed to multiple sexual partners, a phenomenon that is consistent with what has been reported in the literature and linked to early sex initiation (Samkange-Zeeb et al., 2011; Selikow et al., 2009; Sserwanja et al., 2023). Our findings also revealed that individuals in long-term relationships had a lower perception of STI risk, consistent with the literature, which indicates that long, exclusive relationships are associated with a decreased risk of STIs (Mahar & Sherrard, 2024; Sathiyasusuman, 2015). Non-condom use has been linked to committed relationships in previous studies conducted in South Africa (Hendriksen et al., 2007), in which the risk of contracting and transmitting STIs is drastically reduced (Hendriksen et al., 2007; Mokgatle et al., 2021; Samkange-Zeeb et al., 2011). Although South Africa’s SRHR policies are considered the most progressive in the world, SRHR-promoting behaviours among young adults, such as reducing the number of sexual partners and openness regarding HIV status in a relationship, among others, remain a key issue (Lamaro Haintz et al., 2023; Waldman & Stevens, 2015).

Our findings also identified alcohol consumption as a key factor for engaging in risky sexual behaviour, which may be reflective of broader societal alcohol problems in the country (Melesse et al., 2020; Vujovic, Struthers, Meyersfeld, Dlamini, & Mabizela, 2014). A review conducted in South Africa reported that socio-economic and structural characteristics are associated with unfavourable sexual and reproductive outcomes, burdening the country’s youth (Bolarinwa & Boikhutso, 2021). A study in the Eastern Cape, South Africa, reported that alcohol consumption is commonly associated with the risk of contracting and transmitting STIs (Onywera et al., 2022). Another study in the Eastern Cape attributed sexual risk behaviours among 18- to 25-year olds to having sex while intoxicated with alcohol, inconsistent condom use, having multiple partners and others (Mntonintshi-Mketo, Netangaheni, & Lefoka, 2023).

The female participants indicated that they do not have the power to raise their desire to use a condom in a relationship due to the fear of being seen as unfaithful, concurring with the literature (Duby et al., 2021). Thirteen studies included in a narrative systematic review also noted that sex negotiation power is biased towards men, and women who desire condom use are stigmatised as promiscuous (Aventin et al., 2021), as was the case in our study. A study in Uganda also reported that gender norms are structured by a belief system that men have power because they are primary providers in relationships (Stoebenau, Kyegombe, Bingenheimer, Ddumba-Nyanzi, & Mulindwa, 2019). However, a systematic review of articles retrieved from the PubMed search reported that men are as affected by STIs as they have long-term consequences on males’ sexual and reproductive health, including prostatitis, scarring that may lead to infertility and epididymo-orchitis (Henkel, 2021).

Congruent with the literature, we identified several deterrents to SRH service utilisation, including those related to the YFHS, such as healthcare workers’ negative attitudes, lack of privacy, long waiting times, interpersonal relationships with family and friends and financial issues, including the need for transportation to travel to distant places of residence (Amanu et al., 2023; Atuhaire et al., 2021; Holtman et al., 2023; Kyilleh et al., 2018; Mayeye et al., 2010; Ninsiima et al., 2021). Our findings similarly showed that deterrents to SRH service utilisation are directly related to the lack of SRH information and information about the services provided at SRH services (Bolarinwa & Boikhutso, 2021; Mbizvo & Zaidi, 2010).

Our findings suggest that a lack of understanding, knowledge and awareness of SRH services among young adults limits their ability to access them, thereby delaying the realisation of SRHR in the Eastern Cape. A study conducted in Kenya and Zambia reported that 55%–67% of SRH healthcare providers refused to offer SRH services to schoolgirls because they did not approve, a phenomenon consistent with studies conducted in China and Malawi, where healthcare providers also did not approve of SRH services being available to adolescents (Kim Jonas, Crutzen, van den Borne, & Reddy, 2017; Sommer & Mmari, 2015). A systematic review in SSA reported that studies from South Africa, Ethiopia and Uganda reported a scarcity of trained HCWs with accurate SRH information in their countries (Ninsiima et al., 2021). However, a study in Tanzania reported that 37.2% of HCWs reported receiving sufficient SRH information (Ninsiima et al., 2021).

Participants reported that access to SRH information from schools, community outreaches, the media and families enabled them to seek SRH services. Access to SRH information from HCWs has increased SRH service utilisation (Atuhaire et al., 2021). Other reported SRH service utilisation enablers outside health services include supportive and informative parents, supportive peers, sex education in schools, and access to SRH information in mass media (Amanu et al., 2023; Atuhaire et al., 2021; Belay et al., 2021; Mpondo, Ruiter, Schaafsma, van den Borne, & Reddy, 2018; Mwamba et al., 2022; Ninsiima et al., 2021; Vongxay et al., 2019).

### Limitations of the study:

The study excluded individuals under 18 years of age, YFHS non-users and mostly interviewed females, which may limit the transferability of the findings to other contexts. Recruiting participants at the health facility may have created a power imbalance between the participants (patients) and the researcher, a healthcare professional herself, rendering the study prone to social desirability bias. Terms such as SRH, used in the study, may have been technical for lay people, affecting the quality of their responses. The lead female researcher addressed a sensitive subject, which could have caused hesitancy among male participants. However, a thorough introduction and assurances of confidentiality and anonymity mitigated this limitation.

### Study implications:

This study has highlighted a knowledge gap among young adults regarding STIs and SRH. Given knowledge gaps and the fact that young adults did not perceive themselves to be at high risk of contracting and transmitting STIs, tailored health promotion interventions responsive to the needs of young adults are required. Moreover, training materials detailing types of STIs and risks of STIs, including potential premature labour or preterm birth, an increased risk of miscarriage and infertility, the risk of low birth weight, scarring that may lead to infertility in males, prostatitis and the possibility for newborns to have severe eye infections when exposed to STIs, need to be developed for sexual health purposes.

Further research from our study should explore the perceptions of young adults of consultation experience with HCWs who have been trained; the impact of the implemented routine STI screening on the prevalence of STIs; young adults’ perceptions of facilities that offer services during convenient hours; feminisation of STIs; as well as the experiences of young adults with being consulted by younger HCWs.

## Conclusion

The findings from our study suggest a lack of understanding, knowledge and awareness of the link between STIs and SRH among young adults. The knowledge gap in SRH information among young adults negatively influences their intent to engage in safe sexual behaviour and positive SRH service utilisation behaviour. SRH education is needed to empower young adults to make informed decisions about their sexual and reproductive health. However, interventions should be culturally sensitive and socially responsive to the SRH needs of young adults, HCWs, families and peers to handle sexual and reproductive issues properly.

## Data Availability

The datasets generated and analysed during the current study are the property of the University of KwaZulu-Natal (UKZN) and cannot be publicly available. All interested readers can access the dataset from the UKZN Biomedical Research Ethics Committee (BREC) through the following contacts: The Chairperson, Biomedical Research Ethics Administration, Research Office, Westville Campus, Govan Mbeki Building, University of KwaZulu-Natal, P/Bag X54001, Durban, 4000 KwaZulu-Natal, South Africa. Tel.: +27-31-260-4769 Fax: +27-31-260-4609 Email: BREC@ukzn.ac.za.
